# Concurrent metaboreflex activation increases chronotropic and ventilatory responses to passive leg movement without sex-related differences

**DOI:** 10.1007/s00421-023-05186-4

**Published:** 2023-04-04

**Authors:** Fabio Giuseppe Laginestra, Thomas Favaretto, Gaia Giuriato, Camilla Martignon, Chiara Barbi, Anna Pedrinolla, Alessandro Cavicchia, Massimo Venturelli

**Affiliations:** 1 Department of Neurosciences, Biomedicine, and Movement, University of Verona, Verona, Italy; 2 Department of Internal Medicine, University of Utah, 500 Foothill Drive, Salt Lake City, UT 84148 USA; 3 Respiratory Rehabilitation of the Institute of Lumezzane, Istituti Clinici Scientifici Maugeri IRCCS, Lumezzane, Italy

**Keywords:** Mechanoreflex, Metaboreflex, Reflex interaction, Passive leg movement

## Abstract

Previous studies in animal models showed that exercise-induced metabolites accumulation may sensitize the mechanoreflex-induced response. The aim of this study was to assess whether the magnitude of the central hemodynamic and ventilatory adjustments evoked by isolated stimulation of the mechanoreceptors in humans are influenced by the prior accumulation of metabolic byproducts in the muscle. 10 males and 10 females performed two exercise bouts consisting of 5-min of intermittent isometric knee-extensions performed 10% above the previously determined critical force. Post-exercise, the subjects recovered for 5 min either with a suprasystolic circulatory occlusion applied to the exercised quadriceps (PECO) or under freely-perfused conditions (CON). Afterwards, 1-min of continuous passive leg movement was performed. Central hemodynamics, pulmonary data, and electromyography from exercising/passively-moved leg were recorded throughout the trial. Root mean square of successive differences (RMSSD, index of vagal tone) was also calculated. Δpeak responses of heart rate (ΔHR) and ventilation ($\Delta \dot{V}_{{\text{E}}}$) to passive leg movement were higher in PECO compared to CON (ΔHR: 6 ± 5 vs 2 ± 4 bpm, *p* = 0.01; 3.9 ± 3.4 vs 1.9 ± 1.7 L min^−1^, *p* = 0.02). Δpeak of mean arterial pressure (ΔMAP) was significantly different between conditions (5 ± 3 vs  − 3 ± 3 mmHg, *p* < 0.01). Changes in RMSSD with passive leg movement were different between PECO and CON (*p* < 0.01), with a decrease only in the former (39 ± 18 to 32 ± 15 ms, *p* = 0.04). No difference was found in all the other measured variables between conditions (*p* > 0.05). These findings suggest that mechanoreflex-mediated increases in HR and $\dot{V}_{{\text{E}}}$ are sensitized by metabolites accumulation. These responses were not influenced by biological sex.

## Introduction

Cardiovascular and ventilatory adjustments to physical exercise are achieved through the cooperation of different mechanisms. The most important are a feedforward efferent mechanism (i.e., central command), the arterial baroreflex, and the exercise pressor reflex, which is a negative-loop feedback mechanism originating from the working muscle, entailing a mechanosensitive and a metabosensitive branch (Fisher et al. 2015). This feedback from the working muscles is sent to the central nervous system through group III/IV afferent fibers, which convey information about the mechanical distortion of their receptive fields (group III), and metabolic changes happening in the intramuscular milieu (group IV) (Fisher et al. 2015). While the involvement of group IV fibers on hemodynamic regulation is well established (Boushel 2010), the role of mechanosensitive afferent fibers is more difficult to demonstrate. In the last decade, strong evidence for an important role of these fibers in the hemodynamic regulation came using intrathecal fentanyl injection (a potent opioid receptors agonist), which partially blocks afferent feedback to the central nervous system. Indeed, blocking afferent feedback resulted in a substantial decrease in the chronotropic response that typically accompanies passive leg movement (Trinity et al. 2010). Furthermore, using the same pharmacological approach in healthy volunteers (Amann et al. 2010) and a spinal cord injury model, in which afferent feedback is intrinsically interrupted (Venturelli et al. 2012), it has been shown that muscle afferent fibers play a pivotal role also in the ventilatory adjustments to exercise (Amann et al. 2010) and passive movement (Venturelli et al. 2012), respectively.

Classical studies carried out in animal models indicate that metabolic changes in the exercising muscle may sensitize the response to mechanical stress (Rotto et al. 1990; Rotto and Kaufmann 1988). In humans, this issue is more controversial. A previous study utilizing passive stretch of the wrist during post-exercise ischemia, found an increase in the blood pressure response and sympathetic activation (Cui et al. 2008). Similarly, other investigators found a vagally-mediated transient increase in heart rate (HR) when static stretch of the calf muscles was superimposed to circulatory occlusion of the limb (Drew et al. 2008a, b). However, Fisher et al. showed that HR response to static calf stretch was not different after exercise bouts carried out at different intensities, and therefore likely different levels of metabolites accumulation (Fisher et al. 2005). Moreover, since different subsets of group III/IV muscle afferents respond to different stimuli, there is evidence that mechanically-sensitive fibers may be preferentially activated during movement compared to static stretch (Hayes et al. 2005). Therefore, static stretch and dynamic movements may yield different outcomes in terms of autonomic control.

Recently, a study found that when passive cycling was coupled with circulatory occlusion of the lower limbs, chronotropic and ventilatory responses were augmented (Lis et al. 2020). However, as highlighted by Fernandes and Vianna (2020), the accumulation of metabolic byproducts and the presence of electromyographic recordings are of paramount importance to parse out alternative explanations when studying the interaction between metabo- and mechanoreflex.

The aim of this study was to evaluate mechanoreflex-induced central hemodynamic and ventilatory responses when a passive, mechanical stimulation was superimposed over circulatory occlusion compared to when recovery is allowed to take place under freely perfused conditions. Also, our secondary objective was to assess whether a sex-specific response in these assessments was present. Our hypothesis was that mechanoreflex-induced responses would be higher when exercise-induced metabolites were trapped in the muscle by circulatory occlusion and that this outcome would not be different between sexes.

## Methods

### Subjects and ethical approval

Twenty young healthy subjects were recruited for this study (10 males and 10 females, age: 26 ± 3 vs 22 ± 3 years, height: 176 ± 7 vs 163 ± 6 cm, and weight: 76 ± 9 vs 56 ± 7 kg). All subjects were non-smokers and none of them was taking medications as determined by a health questionnaire. They were instructed to report to the lab after having refrained from alcohol and caffeine (≥ 12 h), food (≥ 2 h), and physical exercise (≥ 24 h). The subjects were tested in the same temperature-controlled room (22–24 °C). Moreover, the last experimental session for females was performed during the early follicular phase (days 1–5 from the self-reported menstruation onset) to lessen potential effects of estrogen hormones on hemodynamic responses (Wenner and Stachenfeld 2020). No female participant reported to being using contraceptives at the time of the study. Leg dominance was established based on the self-reported foot used to kick a ball. All subjects but two reported being right-leg dominant. Written informed consent was obtained from each participant after a detailed verbal and written explanation of the experimental procedures. The study complied with the Declaration of Helsinki and was approved by the local ethical committee of the University of Verona (IRB #30444).

### Experimental design and procedures

**Fig. 1:**
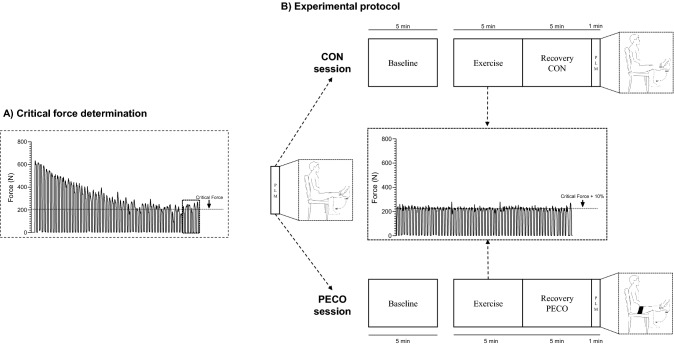
Schematic representation of the study protocol. After the familiarization session (not shown) two sessions were carried out in separate days (**A** and **B**). Critical force (dashed line) in panel **A** was calculated as the mean force expressed in the last six contractions (dashed square). In the second session, post-exercise circulatory occlusion (PECO) or freely perfused (CON) conditions were performed in a counterbalanced manner with a ~ 30-min rest in between. Exercise intensity was set at 10% above critical force (dashed line). *MVC* maximal voluntary contraction; *PLM* passive leg movement

The subjects were asked to report to the lab on three different occasions. On the first experimental day, the subjects were familiarized with the study procedures and with the performance of isometric knee-extensors maximal voluntary contractions (MVC). The instructions for the performance of MVCs were to push “as hard and as fast as possible”, to reach a force plateau in the shortest time possible. A schematic representation of the study protocol is reported in Fig. 1.

On the second experimental day, the subjects performed an all-out isometric knee-extensor test, in order to estimate critical force employing a 60% duty cycle (contraction/relaxation: 3 s/2 s) (Burnley 2009). The 5 min all-out test consisted of 60 knee-extension MVCs using the same duty cycle abovementioned. During the test, participants were verbally encouraged by the members of the research team to ensure maximal effort. The subjects were seated with a 90° knee flexion on a custom-built chair, with their ankle linked to the force transducer and a steel bar through a noncompliant strap, which was placed 2-cm above the lateral malleolus of the ankle. Extraneous movement of the upper body was avoided by two-crossed belts over the chest while the hips were stabilized by an additional belt. An audio recording signaled the start and stop of each contraction and the subjects were able to see the force feedback on a wall projected ~ 3 m in front of them. No information was given to the subjects concerning the time elapsed or remaining. Critical force was calculated as the average of the mean force exerted during the last six contractions (Burnley 2009).

On the third and last experimental session, participants were instrumented and allowed to rest in the sitting position for 15 min. After this resting period, a 1-min baseline measurement was collected before the subject’s lower leg was passively moved for 1 min at 1 Hz. Afterwards, they were asked to perform two identical exercise bouts in which the recovery was done either without (CON) or with post-exercise circulatory occlusion (PECO). Circulatory occlusion was obtained by inflating a tourniquet cuff (DTC-506. Daesung Maref, South Korea) around the proximal part of the exercising thigh at a suprasystolic pressure (300 mmHg) with a custom-made rapid cuff occlusion system (< 0.5 s to full occlusion). These two bouts were interspersed by a 30 min rest and were carried out in a counterbalanced manner to minimize any eventual carry-over effect. Each bout started with a 5-min baseline period. Then, a MVC was performed to normalize EMG signals. After an adequate pause (~ 1 min) allowing the hemodynamic values to return to baseline, 5 min of rhythmic isometric knee-extension (3 s on/2 s off) were performed at an intensity that was 10% above critical force, which was determined in the previous experimental visit. This intensity has been chosen because it has been shown to be adequate to induce significant metabolic perturbation in the exercising muscle (Jones et al. 2008; Burnley et al. 2012). At the end of the exercise, the subjects started the 5-min recovery period either with or without PECO. Finally, the lower leg of the subjects was again passively moved for 1 min at a frequency of 1 Hz. All passive movement procedures were performed by the same member of the research team, moving the subjects’ lower leg through the range of motion defined by 90 and 180° knee joint angles (where the fully extended knee joint is defined as 180°).

### Central hemodynamics and pulmonary data

Blood pressure, cardiac output (CO), and stroke volume (SV) were measured on a beat-by-beat basis using a finger photoplethysmography device (Finapres model 2300; Ohmeda, Englewood, CO, USA). The left hand was held on a custom-made support, at the level of the heart and a photoplethysmographic cuff was placed on the fourth finger. A software extension (Non-Invasive Cardiac Output, ADInstruments, Australia) was added to the LabChart 8 software to apply the Modelflow algorithm to the raw beat-by-beat data from the non-invasive blood pressure measurement device. Mean arterial pressure (MAP) was calculated as diastolic blood pressure + 1/3 (systolic − diastolic blood pressure). HR was calculated beat-by-beat from the electrocardiographic signal collected with a dual bioamplifier (ML135, ADInstruments, Australia) at 2 kHz.

Pulmonary gas exchange ($\dot{V}{\text{O}}_{{2}}$ and $\dot{V}{\text{CO}}_{{2}}$) minute ventilation ($\dot{V}_{{\text{E}}}$), breathing frequency (*f*_B_) and tidal volume (*V*_T_) were measured breath-by-breath with a metabolic cart (Quark b^2^, Cosmed, Italy). Before each session, after an appropriate warm-up, the gas analyzer and the turbine flowmeter were calibrated according to the instructions of the manufacturer.

### Isometric force, surface electromyography, and rating of perceived exertion

Isometric force was measured by a force transducer (model UU2; DaCell, Korea) previously calibrated, connected to a custom-made chair through a noncompliant strap placed around the subjects’ ankle. The output from the force transducer was amplified and filtered with a 20 Hz low-cut filter and recorded at a sampling rate of 2 kHz.

Vastus lateralis electromyography (EMG) was continuously recorded with a dual bioamplifier (ML135, ADInstruments, Australia). Two surface Ag/AgCl electrodes (PG10C; Fiab, Vicchio, Florence, Italy) were attached to the skin with a 20-mm inter-electrode distance. The electrodes were placed longitudinally, in line with the underlying muscle fibers arrangement, at two-thirds of the distance between the anterior iliac spine and the lateral part of the patella (Hermens et al. 2000). Before the application of the electrodes, the skin was shaved, abraded with sandpaper, and finally cleaned with an alcohol swab in order to minimize skin impedance. The raw EMG signal was amplified and digitized online at a 2 kHz sampling frequency. Acquisition of the EMG data was done using a computer-based data acquisition and analysis system (hardware: PowerLab 16/30; ML880, ADInstruments, Bellavista, Australia and software: LabChart 8, ADInstruments, Bellavista, Australia). Finally, rating of perceived exertion (RPE) was obtained at every minute using the 6–20 Borg scale (Borg 1975).

### Data analysis

All central hemodynamics and breath-by-breath data were linearly interpolated to 1-s intervals and time-aligned to the onset of passive leg movement. Successively, data from the 30 s before passive leg movement was averaged and represented baseline values. Pulmonary data was checked visually for eventual aberrant breaths. When an aberrant breath was found, it was eliminated from the analysis and data from the two adjacent breaths were linearly interpolated. All hemodynamics and pulmonary data were smoothed using a 3-s rolling average. Statistical analysis for the passive leg movement-induced responses was performed on the Δpeak values from baseline (30 s before the onset of passive leg movement).

Root mean square of successive differences (RMSSD) was calculated from the R–R intervals of the electrocardiogram during the 60-s period preceding the onset of the passive movement and the first 15 s afterwards. This time point was chosen because changes in vagal tone may be expected here based on previous literature (Drew et al. 2008a). RMSSD is a recommended time-domain measure of short-term HR variability and it is sensitive to changes in vagal tone (Task-Force 1996) and relatively free of respiratory influences (Hill and Siebenbrock 2009). RMSSD data from one subject was removed from the analysis because it was > 5 SD compared to the average data.

EMG data were analyzed with an in-house built MATLAB routine (MATLAB 2020b, Mathworks, USA). The raw EMG signal was bandpass filtered (10–500 Hz) with a fourth order, zero-phase, Butterworth filter and full-wave rectified. For the exercise EMG, a 500 ms baseline was detected between contractions, and onsets were set when the signal rose by > 3SD from baseline values. The same algorithm was applied to find contraction offset. For each muscle contraction, the root mean square (RMS) was calculated and normalized by the highest 500 ms EMG_RMS_ obtained during a MVC performed before exercise (Laginestra et al. 2022). Moreover, the EMG_RMS_ of the 30 s period preceding passive leg movement, and the 60 s of passive leg movement were calculated to ensure that the subjects were not voluntarily contracting the muscles of the passively moved limb.

### Statistical analysis

Two-tailed independent sample *t*-tests were employed for baseline MVC, critical force, and exercise intensity between females and males. Paired samples *t*-tests were used between rest and passive leg movement within each condition for EMG measurements. Also, a three-way (condition × time × sex) ANOVA for repeated measures was performed to find eventual differences between conditions during the three phases of the protocol for all the cardiorespiratory variables (rest–exercise–recovery) and for RMSSD (baseline–movement). Successively, a two-way (condition × sex) ANOVA for repeated measures was performed to employed to find differences in the Δpeak responses to passive leg movement between PECO and CON. If significant interactions were found, pairwise differences were identified using Bonferroni post-hoc test correction for multiple comparisons when appropriate. Statistical analysis was performed with IBM SPSS Statistics 24 (IBM Corp©, 2016) and figures were made with GraphPad Prism 8.0 (GraphPad Software, Inc., 2012). Significance level was set at α < 0.05 and effect sizes were reported by calculating Cohen’s *d* and partial eta squared (η_p_^2^). Data are expressed as mean ± SD unless otherwise stated.

## Results

### Maximal voluntary contraction, critical force, and exercise intensity

In our participants, MVC was 595 ± 158 N (females: 473 ± 81 N; males: 718 ± 113 N, *t*_18_ = −5.6, *p* < 0.01, *d* = 2.49). Critical force was 195 ± 57 N (females: 159 ± 22 N; males: 230 ± 59 N, *t*_18_ = −3.6, *p* < 0.01, *d* = 1.59), which was equivalent to 33 ± 8% MVC (females: 34 ± 7%; males: 32 ± 9%, *t*_18_ = 0.5, *p* = 0.17, *d* = 0.25). The exercise intensity used for PECO and CON was 214 ± 62 N (females: 175 ± 24 N; males: 253 ± 65 N, *t*_18_ = −3.6, *p* < 0.01, *d* = 1.59). which was equivalent to 37 ± 9% MVC (females: 38 ± 8%; males: 36 ± 10%, *t*_18_ = 0.8, *p* = 0.62, *d* = 0.22).

### Central hemodynamics and root mean square of successive differences

Rest, exercise, and recovery data for the two conditions are presented in Table 1. All variables were similar between sexes and conditions at rest and during exercise (all *p* > 0.05). During recovery after exercise, HR, SV, and CO returned to baseline in both conditions, while MAP remained elevated during PECO.

**Table 1:** Baseline, exercise, and recovery data for cardiovascular and respiratory variables

		Baseline	Exercise	Recovery
HR (bpm)	CON	78 ± 13	96 ± 19*	75 ± 11*
	PECO	77 ± 11	97 ± 18*	77 ± 14*
SV (mL)	CON	71 ± 18	79 ± 17	69 ± 18
	PECO	72 ± 15	76 ± 16	67 ± 16
CO (L·min^−1^)	CON	5.5 ± 1.2	7.5 ± 1.4*	5.1 ± 1.0*
	PECO	5.4 ± 1.2	7.2 ± 1.4*	5.1 ± 1.0*
MAP (mmHg)	CON	91 ± 6	112 ± 9*	92 ± 7^*,§^
	PECO	91 ± 7	112 ± 9*	103 ± 8^*,§^
$\dot{V}_{{\text{E}}}$(L·min^−1^)	CON	14.8 ± 4.5	21.6 ± 6.6*	10.4 ± 2.5*
	PECO	13.5 ± 3.5	20.6 ± 5.3*	11.4 ± 4.8*
$\dot{V}{\text{O}}_{2}$(mL·kg^−1^·min^−1^)	CON	6.6 ± 1.6	10.0 ± 2.0*	4.6 ± 0.8*
	PECO	6.3 ± 1.4	10.1 ± 2.3*	4.5 ± 0.9*
$\dot{V}{\text{CO}}_{2}$(mL·kg^−1^·min^−1^)	CON	5.7 ± 1.7	9.1 ± 2.3*	3.8 ± 0.7*
	PECO	5.3 ± 1.2	9.0 ± 2.4*	4.0 ± 1.2*
$\dot{V}_{{\text{E}}} /\dot{V}{\text{CO}}_{2}$	CON	37.2 ± 4.6	34.1 ± 3.5*	37.2 ± 4.8*
	PECO	36.6 ± 4.5	33.1 ± 3.2*	38.5 ± 6.5*

*HR* heart rate; *SV* stroke volume; *CO* cardiac output; *MAP* mean arterial pressure; $\dot{V}_{{\text{E}}}$ minute ventilation; $\dot{V}{\text{O}}_{2}$ oxygen consumption; $\dot{V}{\text{CO}}_{2}$ carbon dioxide production $\dot{V}_{{\text{E}}} /\dot{V}{\text{CO}}_{2}$ ventilatory equivalent for CO_2_; *CON* control; *PECO* post-exercise circulatory occlusion

*Significantly different from previous time point (*p* < 0.05); ^§^significantly different from the other condition (*p* < 0.05). *n* = 20

**Fig. 2:**
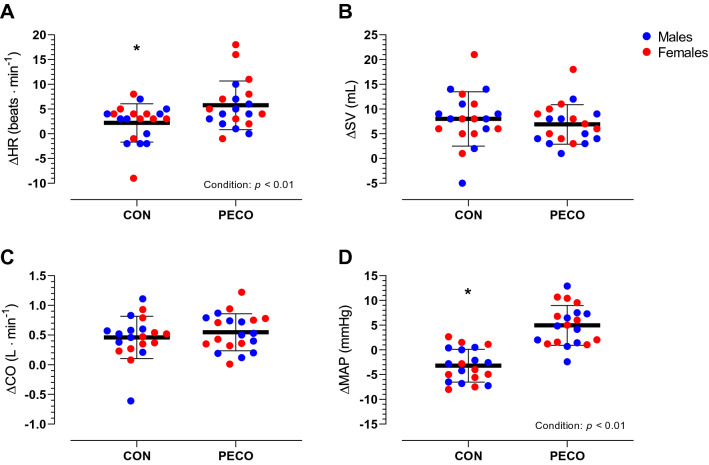
Passive leg movement-induced peak changes in central hemodynamics variables with post-exercise circulatory occlusion (PECO) or freely perfused recovery (CON). *Significantly different than PECO. **A** *HR* heart rate; **B** *SV* stroke volume; **C** *CO* cardiac output; **D** *MAP* mean arterial pressure. Statistical significance was set at *p* <0.05. Number of participants (*n*) = 20

Δpeak values for central hemodynamics variables during passive leg movement are presented in Fig. 2. There was no statistically significant difference in any cardiovascular variable between Δpeak responses observed in CON and the passive leg movement performed at the beginning of the session (all *p* > 0.05). No significant sex effect (*F*_1,18_ = 1.1, *p* = 0.31, η_p_^2^ = 0.06) nor condition × sex interaction was found in HR (*F*_1,18_ = 1.7, *p* = 0.21, η_p_^2^ = 0.09). However, a significant difference in HR behavior was found between conditions (*F*_1,18_ = 11.5, *p* < 0.01, η_p_^2^ = 0.39) whereby HR increased by ~ 3% in CON (75 ± 11 to 78 ± 12 bpm) and ~ 8% in PECO (77 ± 14 to 83 ± 17 bpm). Furthermore, the change in SV was similar between conditions (CON: 69 ± 18 to 77 ± 19 mL, PECO: 67 ± 15 to 74 ± 16 mL, *F*_1,18_ = 0.5, *p* = 0.48, η_p_^2^ = 0.03) and between sexes (sex: *F*_1,18_ = 1.1, *p* = 0.31, η_p_^2^ = 0.06; condition × sex interaction: *F*_1,18_ = 0.3, *p* = 0.60, η_p_^2^ = 0.02). However, together these adjustments did not translate into a different increase in CO (CON: 5.08 ± 0.98 to 5.54 ± 1.07 L·min^−1^, PECO: 5.05 ± 1.00 to 5.60 ± 1.07 L·min^−1^, *F*_1,18_ = 0.7, *p* = 0.40, η_p_^2^ = 0.04) nor between sexes (sex: *F*_1,18_ = 1.0, *p* = 0.76, η_p_^2^ = 0.01; condition × sex interaction: *F*_1,18_ = 0.2, *p* = 0.66, η_p_^2^ = 0.01). A divergent response in MAP (*F*_1,18_ = 50.8, *p* < 0.01, η_p_^2^ = 0.74) was found during passive leg movement in CON (92 ± 7 to 89 ± 9 mmHg) and in PECO (103 ± 8 to 108 ± 9 mmHg). No sex difference was found in MAP (sex: *F*_1,18_ = 0.1, *p* = 0.75, η_p_^2^ = 0.01; condition × sex interaction: *F*_1,18_ = 0.2, *p* = 0.65, η_p_^2^ = 0.01) (Fig. 2).

**Fig. 3:**
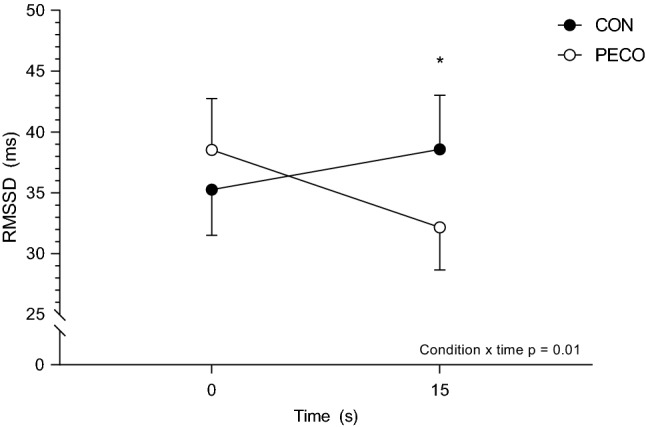
Root mean square of successive differences (RMSSD) from baseline to 15 s after the onset of the passive leg movement. *Significantly different than the previous time point in PECO. Statistical significance was set at *p* < 0.05. Data are presented mean ± SEM. Number of participants (*n*) = 19

No significant three-way interaction was found for RMSSD (condition × time × sex: *F*_1,17_ = 1.0, *p* = 0.33, η_p_^2^ = 0.06). On the same note, no significant condition × sex (*F*_1,17_ = 1.8, *p* = 0.20, η_p_^2^ = 0.01) or time × sex (*F*_1,17_ = 3.3, *p* = 0.09, η_p_^2^ = 0.16) interactions were detected. On the other hand, a significant condition × time interaction was found in RMSSD (*F*_1,17_ = 13.2, *p* < 0.01, η_p_^2^ = 0.44, Fig. 3). Follow-up pairwise comparison showed that RMSSD during the first 15 s of passive leg movement was different than baseline in PECO only (39 ± 18 to 32 ± 15 ms, *p* = 0.04, *d* = 0.42).

### Pulmonary variables

Rest, exercise, and recovery data for the variables of interest are presented in Table 1.

**Fig. 4:**
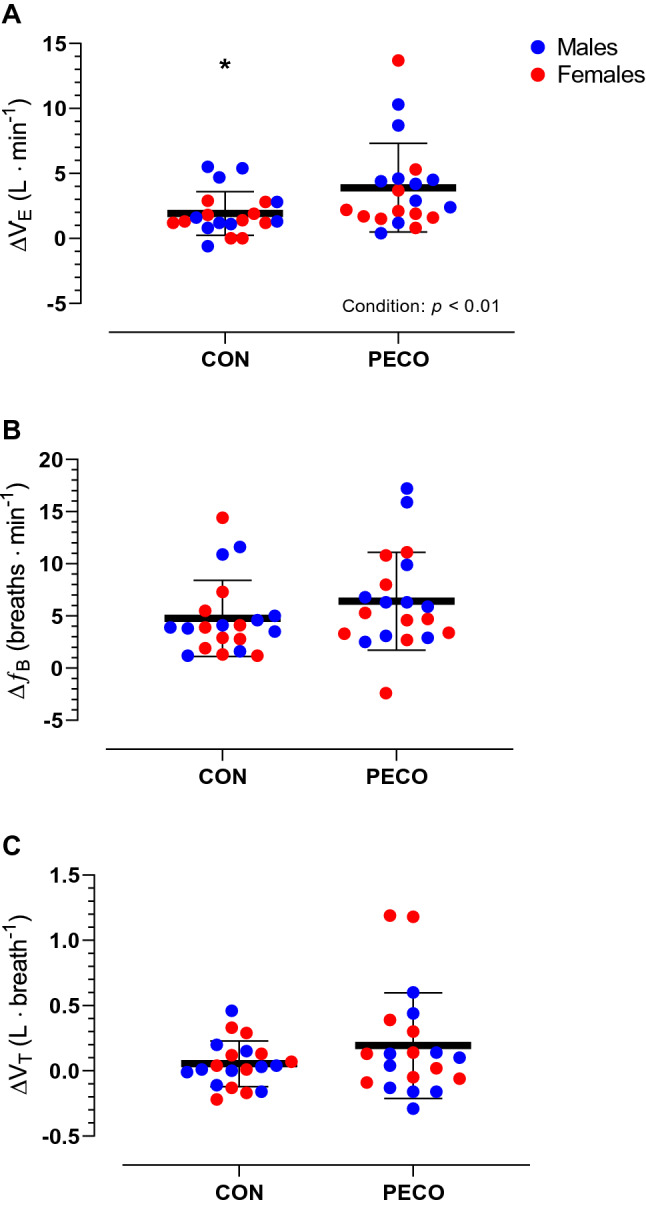
Passive leg movement-induced peak changes in in ventilatory variables during passive leg movement with post-exercise circulatory occlusion (PECO) or freely perfused recovery (CON). *Significantly different than PECO. **A** $\dot{V}_{{\text{E}}}$: minute ventilation; **B** *f*_B_: breathing frequency; **C** V_T_: tidal volume. Statistical significance was set at *p* < 0.05. Number of participants (*n*) = 20

Furthermore, Δpeak values for ventilatory variables in response to passive leg movement are presented in Fig. 4. There was no statistically significant difference in any ventilatory variable between Δpeak responses observed in CON and the passive leg movement performed at the beginning of the session (all *p* > 0.05). During passive leg movement, Δpeak for $\dot{V}_{{\text{E}}}$ increased by ~ 40% in PECO (11.4 ± 4.8 to 15.3 ± 5.9 L·min^−1^) and only ~ 20% in CON (10.4 ± 2.5 to 12.3 ± 2.9 L·min^−1^) with this difference being statistically significant between conditions (condition: *F*_1,18_ = 5.6, *p* = 0.03, η_p_^2^ = 0.24). No sex (*F*_1,18_ = 1.1, *p* = 0.31, η_p_^2^ = 0.06), nor condition × sex interaction was found (*F*_1,18_ = 0.0, *p* = 0.99, η_p_^2^ = 0.00). Concurrently, the increase in *V*_T_ was not significantly different (*F*_1,18_ = 2.4, *p* = 0.15, η_p_^2^ = 0.12) between CON (0.72 ± 0.28 to 0.77 ± 0.29 L) and PECO (0.87 ± 0.40 to 1.06 ± 0.52 L) nor between sexes (sex: *F*_1,18_ = 1.2, *p* = 0.28, η_p_^2^ = 0.06; condition × sex: *F*_1,18_ = 2.0, *p* = 0.17, η_p_^2^ = 0.10). Finally, also Δpeak response in *f*_B_ was not different (*F*_1,18_ = 1.6, *p* = 0.23, η_p_^2^ = 0.08) between CON (16.4 ± 5.2 to 21.2 ± 5.2 breaths·min^−1^) and PECO (14.8 ± 5.2 to 21.2 ± 5.3 breaths·min^−1^) and between sexes (sex: *F*_1,18_ = 1.3, *p* = 0.28, η_p_^2^ = 0.07; condition × sex: *F*_1,18_ = 0.6, *p* = 0.45, η_p_^2^ = 0.03).

### Electromyography and rate of perceived exertion

**Fig. 5:**
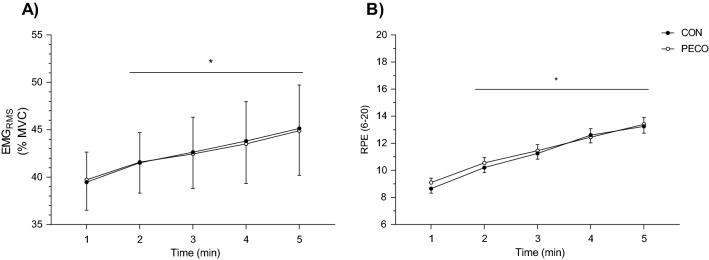
**A** Electromyography and **B** rating of perceived exertion (RPE) during intermittent isometric knee-extensions performed 10% above critical force. Since no differences were found between conditions, data were pooled to display the effect of time. *Significantly different than the previous time point. Statistical significance was set at *p*  0.05. Data are presented as mean ± SD. Number of participants (*n*) = 20

EMG activity during passive leg movement was not different from the preceding resting period in both conditions CON (2.40 ± 1.90% to 2.40 ± 1.91%, *p* = 0.93, *d* = 0.24) or PECO (2.35 ± 1.89% to 2.37 ± 1.87%, *p* = 0.30, *d* = 0.24). EMG_RMS_ and RPE during exercise are presented in Fig. 5. EMG_RMS_ increased over time in both conditions (pooled values from *min 1* to *min 5*: 39.6 ± 13.0% to 45.0 ± 20.5%, *p* < 0.01, η_p_^2^ = 0.18), with no difference between CON and PECO (*p* = 0.97, η_p_^2^ = 0.01). Also, RPE demonstrated the same behavior by increasing over time (pooled values from *min 1* to *min 5:* 9 ± 2 to 13 ± 2, *p* < 0.01, η_p_^2^ = 0.75) with no difference between conditions (*p* = 0.33, η_p_^2^ = 0.06).

## Discussion

The primary objective of this investigation was to study whether the central hemodynamic and ventilatory adjustments to passive leg movement (i.e., mechanoreflex) interact with the effects caused by intramuscular metabolites accumulation (i.e., metaboreflex). The main findings of this study were that chronotropic (ΔHR) and ventilatory ($\Delta \dot{V}_{{\text{E}}}$) responses to passive leg movement were higher in both sexes when the previously exercised muscle was maintained ischemic compared to a situation in which the muscle was freely perfused. These higher responses were accompanied by a larger decrease in the vagal tone at the onset of the passive movement. This finding supports the concept that the cardiorespiratory response to mechanical stimulation, is potentiated when metabolites are accumulated in the muscle and suggest that this phenomenon is mediated by a transient decrease in the parasympathetic drive to the heart, and likely an increase in the overall sympathetic activation.

### The effects of combined metabo- and mechanoreflex activation on central hemodynamics

Whether an interaction between the mechanical and metabolic branches of muscle afferents exists in humans is still controversial with studies demonstrating an influence (Cui et al. 2008; Lis et al. 2020; Bell and White 2005; Nishiyasu et al. 2006) and others demonstrating no influence (Fisher et al. 2005; Venturelli et al. 2017). In humans, isolated muscle mechanoreceptors stimulation through passive stretch (Gladwell et al. 2005; Gladwell and Coote 2002), and dynamic movement (Venturelli et al. 2017; McDaniel et al. 2010) has been shown to be able to evoke a transient HR and blood pressure response (Cui et al. 2006). Given the rapidity of this response, and the fact that infusion of anticholinergic drugs (i.e., glycopyrrolate) abolishes it, this chronotropic adjustment is attributed to an effect on vagal withdrawal (Gladwell et al. 2005).

Our results showed a transient but significantly higher increase in HR response (~ 8% vs. ~ 3%) to passive leg movement when ischemia was maintained on the moved leg after exercise. However, this difference was too small to result in a higher CO response compared to CON, limiting its functional importance in this model. Importantly, the results from EMG data suggest that there was no involvement of central command during the passive leg movement and indicate that the observed effect is due to a reflex mechanism. Interestingly, we also observed a significant decrease in RMSSD only in PECO in response to passive leg movement (Fig. 3). This result agrees with the results of a previous study in which static stretch superimposed on PECO transiently decreased RMSSD (Drew et al. 2008a) and supports the idea that the mechanism between the mechano- and metaboreflex interaction, may be represented by a transient decrease of the vagal tone during the first seconds after movement onset. Therefore, the sensitization of group III fibers, together with the sympathoexcitation due to higher group IV afferents firing, may be a sufficient stimulus to temporarily increase HR and MAP. In fact, previous studies showed how this interaction may lower baroreflex sensitivity and, therefore, its ability to control changes in HR (Drew 2017; Drew et al. 2008a). The observation that HR was not different between PECO and CON during recovery (Table 1) agrees with previous studies showing that PECO alone does not cause enough sympathoexcitation to override the parasympathetic reactivation happening with the cessation of central command (Nishiyasu et al. 1994; Iellamo et al. 1999). Finally, a recent study by Peçanha and colleagues showed that activation of the mechanoreflex during post-exercise plays a role in attenuating heart rate recovery, highlighting the importance of these mechano-sensitive fibers on the cardiac parasympathetic branches of the autonomic nervous system (Pecanha et al. 2021).

### The effects of combined metabo- and mechanoreflex activation on ventilatory drive

The role of muscle afferents stimulation on ventilatory responses is not univocal with previous studies using various approaches, yielding inconsistent results, also dependent on the modality to stimulate group III/IV afferents. For example, passive calf stretch leads to a non-significant increase in $\dot{V}_{{\text{E}}}$ (Bruce and White 2012), while bilateral passive leg movement has been found to provoke a ~ 6 L·min^−1^ increase in $\dot{V}_{{\text{E}}}$ (Bell and Duffin 2006). A significant increase in ventilation was also found when external pulsed muscle compressions were superimposed on rhythmic exercise (Nishiyasu et al. 2006). However, while the use of large muscle masses may be more appropriate because of the evidence that the magnitude of afferent feedback is related to the size of the involved muscle mass (Iwamoto and Botterman 1985) it also makes the contribution of central command harder to parse out, given the possible involvement of postural, stabilizing muscles, not directly involved in the passive movement.

In this study, we found that passive movement of a single leg with PECO was accompanied by an increase in peak $\Delta \dot{V}_{{\text{E}}}$ that was almost twofold the one observed during CON. The fact that neither Δ*V*_T_ nor Δ*f*_B_ were significantly different between PECO and CON may suggest that neither factor alone is responsible for the increased response in $\dot{V}_{{\text{E}}}$ in this model, but that may be the result of changes in both variables, which are known to be regulated following distinct inputs (Forster et al. 2012). This larger increase in $\dot{V}_{{\text{E}}}$ during the combined activation of metabolically and mechanically sensitive afferents, may signify that the effects of the two pathways are interactive. In fact, while it is well accepted that post-exercise ischemia is unable to sustain ventilatory drive in and of itself (Bruce et al. 2019), evidence from recent studies suggests that inputs from different regulatory mechanisms (e.g. central command, chemoreflexes) need to act synergistically in order to increase the ventilatory responses to muscle afferents stimulation (Silva et al. 2018; Wan et al. 2020; Lam et al. 2019). In an elegantly designed study, Lam and colleagues recently showed that when PECO was superimposed to the previously exercised contralateral leg, the ventilatory response to a successive exercise bout (i.e., involving central command) was accentuated, with the “excess” response attributable to enhanced afferent firing (Lam et al. 2019). Accordingly, a study from Silva et al., demonstrated that when the subjects were breathing a hypoxic mixture, the ventilatory response to passive leg movement was amplified compared to the passive movement alone, demonstrating an interaction when the chemoreflex and the mechanoreflex were stimulated together (Silva et al. 2018).

The mechanism behind the increase in ventilation in our study is not clear. However, since ventilation is not controlled by parasympathetic innervation, we speculate that our findings may be the result of a concomitant sympathetic activation caused by the stimulation of group III afferents (Victor et al. 1989).In support of this idea, a previous study from our group demonstrated how passive sympathoexcitatory maneuvers (e.g., static stretching) are able to restrain the peripheral hyperemic response to a passively moved, remote limb, likely through sympathetic vasoconstriction (Zambolin et al. 2022).

### Absence of sex-related differences in mechanoreflex-induced central responses

In the present study, an exploratory analysis was conducted in order to assess whether any of the central responses to the mechano-metaboreflex interaction, presented sex-related differences. Our findings did not uncover any sex-specific responses in cardiac or ventilatory responses, indicating that the males and females similarly respond to the interaction of the two branches of the group III/IV muscle afferent pathway. To our knowledge, this is the first study to provide empirical evidence about this issue. Even though the literature is scarce, a previous study found that the isolated mechanoreflex activation through passive leg movement resulted in a blunted central hemodynamic response (i.e., lower HR) in healthy young females compared to their male counterparts (Ives et al. 2013). On the other hand, similar to the present study, a recent investigation from Wan and colleagues (Wan et al. 2022), found that when passive leg movement was performed in isolation, HR response was similar between sexes. Interestingly however, biological sex had an important effect when the mechanoreflex acted in concert with the chemoreflex, highlighting, once again, the importance of studying the interaction between reflexes instead of their isolated effects. On this note, further research is needed to evaluate how biological sex and reflexes interact in the neural control of autonomic adjustments. Finally, although the sample size employed in the present study is relatively close to the ones used in previous investigations addressing similar experimental questions (Ives et al. 2013; Wan et al. 2022), future studies should consider increasing the number of participants for each sex to ensure adequate statistical power.

### Experimental considerations

In the present study, the activation of the metaboreflex was performed by inflating a cuff at suprasystolic pressure (standardized at 300 mmHg for all participants). It is important to point out that this procedure is usually associated with significant pain. Since noxious stimulation is also effective in activating group IV afferents (Pollak et al. 2014) our results are likely representing the response of metabo- and nociceptors.

It may be argued that performing passive leg movement after 5 min of recovery from exercise in CON may have an effect on the observed responses for two reasons: (1) 5 min may not be enough to washout all the metabolic byproducts of exercise, and (2) given the proximity of the passive movement to exercise, this may be influenced by other factors such as arousal, defined as an increase in brain activity independent of motor command (Bell and Duffin 2004; Venturelli et al. 2012). To verify this proposition, we compared the responses observed in CON with the ones obtained during the passive leg movement performed at the beginning of each experimental session (Fig. 1, panel B). Since there were no differences in any of the studied variables between the two trials, these results support the idea that full recovery was achieved and that the observed mechanoreflex-induced responses were likely not influenced by arousal.

In this study, we decided to use the critical intensity model to establish the exercise intensity for our protocol, instead of basing it on a fixed %MVC. To the best of our knowledge, this approach is novel in the study of autonomic adjustments to metabolic and mechanical stimulation. Our choice stems from the observation that metabolites accumulation depends on the metabolic domain in which exercise is performed (Jones et al. 2008). In fact, given the high inter-subjects variability in the levels of %MVC at which critical intensity is located (Kellawan and Tschakovsky 2014; Burnley 2009), a fixed %MVC would likely create very different metabolic perturbations, which could be extremely high for a subject or extremely low for another (Kent-Braun et al. 1993). Accordingly, a study using ^31^P magnetic resonance spectroscopy demonstrated how the kinetics of changes in the intramuscular metabolic milieu differ when exercise is performed above, or below critical intensity (Jones et al. 2008). Also, it is becoming increasingly recognized that absolute muscle strength influences the pressor response to exercise (Lee et al. 2021; Notay et al. 2018), which may also partly be explained by this proposition. In fact, the levels of blood flow occlusion caused by higher absolute forces would, in turn, cause longer time under ischemia and different rates of metabolites accumulation. Therefore, we believe that the approach used in our study is more suitable to compare physiological responses that are highly dependent on metabolites accumulation, and we advocate for future studies to apply this concept to further investigate the role of intensity domains on autonomic adjustments to exercise, especially when between-subjects designs are employed.

## Conclusions

In conclusion, in this study we have shown that mechanoreflex-induced cardiac and ventilatory responses to passive leg movement are sensitized by the metabolic conditions of the muscle in young adults, independently from biological sex. Our data suggest that a transient decrease in vagal tone and a likely concurrent increase in sympathetic activation mediate the increase in chronotropic and ventilatory responses when passive leg movement is superimposed on metaboreflex activation.

## Data Availability

The datasets generated in the current study are available from the corresponding author upon reasonable request.
