# Correction to: Concurrent metaboreflex activation increases chronotropic and ventilatory responses to passive leg movement without sex‑related differences

**DOI:** 10.1007/s00421-023-05203-6

**Published:** 2023-05-03

**Authors:** Fabio Giuseppe Laginestra, Thomas Favaretto, Gaia Giuriato, Camilla Martignon, Chiara Barbi, Anna Pedrinolla, Alessandro Cavicchia, Massimo Venturelli

**Affiliations:** 1 Department of Neurosciences, Biomedicine, and Movement, University of Verona, Verona, Italy; 2 Department of Internal Medicine, University of Utah, 500 Foothill Drive, Salt Lake City, UT 84148 USA; 3 Respiratory Rehabilitation of the Institute of Lumezzane, Istituti Clinici Scientifici Maugeri IRCCS, Lumezzane, Italy

**Correction to: European Journal of Applied Physiology** 10.1007/s00421-023-05186-4

The original version of this article unfortunately contained a mistake. Both Figs. 3 and 4 are the same.

The Fig. 3 should have appeared as shown below.

**Fig. 3:**
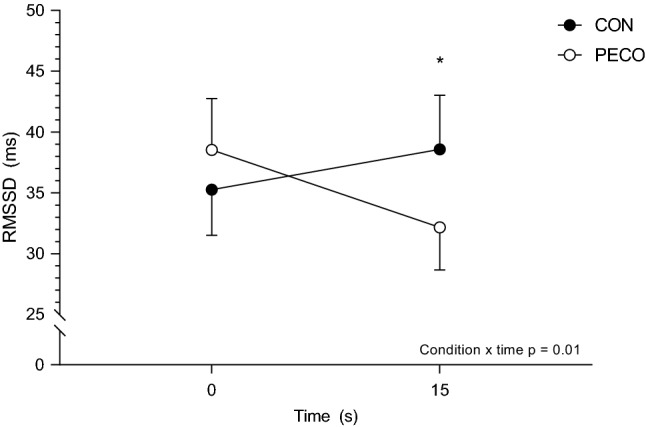
Root mean square of successive differences (RMSSD) from baseline to 15 s after the onset of the passive leg movement. *Significantly different than the previous time point in PECO. Statistical significance was set at *p* < 0.05. Data are presented mean ± SEM. Number of participants (*n*) = 19

The original article has been corrected.

